# HR-pQCT cross-calibration using standard vs. Laplace-Hamming binarization approach

**DOI:** 10.1093/jbmrpl/ziae116

**Published:** 2024-08-27

**Authors:** Saghi Sadoughi, Aditya Subramanian, Gabriella Ramil, Minhao Zhou, Andrew J Burghardt, Galateia J Kazakia

**Affiliations:** Bone Quality Research Lab, Department of Radiology and Biomedical Imaging, University of California, San Francisco, 185 Berry St., Suite 350, San Francisco, CA 94107, United States; Bone Quality Research Lab, Department of Radiology and Biomedical Imaging, University of California, San Francisco, 185 Berry St., Suite 350, San Francisco, CA 94107, United States; Bone Quality Research Lab, Department of Radiology and Biomedical Imaging, University of California, San Francisco, 185 Berry St., Suite 350, San Francisco, CA 94107, United States; Bone Quality Research Lab, Department of Radiology and Biomedical Imaging, University of California, San Francisco, 185 Berry St., Suite 350, San Francisco, CA 94107, United States; Bone Quality Research Lab, Department of Radiology and Biomedical Imaging, University of California, San Francisco, 185 Berry St., Suite 350, San Francisco, CA 94107, United States; Bone Quality Research Lab, Department of Radiology and Biomedical Imaging, University of California, San Francisco, 185 Berry St., Suite 350, San Francisco, CA 94107, United States

**Keywords:** HR-pQCT, Laplace-Hamming binarization, Gaussian binarization, cross-calibration, estimation error

## Abstract

High-resolution peripheral quantitative computed tomography (HR-pQCT) has emerged as a powerful imaging technique for characterizing bone microarchitecture in the human peripheral skeleton. The second-generation HR-pQCT scanner provides improved spatial resolution and a shorter scan time. However, the transition from the first-generation (XCTI) to second-generation HR-pQCT scanners (XCTII) poses challenges for longitudinal studies, multi-center trials, and comparison to historical data. Cross-calibration, an established approach for determining relationships between measurements obtained from different devices, can bridge this gap and enable the utilization and comparison of legacy data. The goal of this study was to establish cross-calibration equations to estimate XCTII measurements from XCTI data, using both the standard and Laplace-Hamming (LH) binarization approaches. Thirty-six volunteers (26–85 yr) were recruited and their radii and tibiae were scanned on both XCTI and XCTII scanners. XCTI images were analyzed using the manufacturer’s standard protocol. XCTII images were analyzed twice: using the manufacturer’s standard protocol and the LH segmentation approach previously developed and validated by our team. Linear regression analysis was used to establish cross-calibration equations. Results demonstrated strong correlations between XCTI and XCTII density and geometry outcomes. For most microstructural outcomes, although there were considerable differences in absolute values, correlations between measurements obtained from different scanners were strong, allowing for accurate cross-calibration estimations. For some microstructural outcomes with a higher sensitivity to spatial resolution (eg, trabecular thickness, cortical pore diameter), XCTII standard protocol resulted in poor correlations between the scanners, while our LH approach improved these correlations and decreased the difference in absolute values and the proportional bias for other measurements. For these reasons and due to the improved accuracy of our LH approach compared with the standard approach, as established in our previous study, we propose that investigators should use the LH approach for analyzing XCTII scans, particularly when comparing to XCTI data.

## Introduction

High-resolution peripheral quantitative computed tomography (HR-pQCT) has emerged as a powerful non-invasive imaging technique for in vivo characterization of bone microarchitecture in the human peripheral skeleton. HR-pQCT quantifies geometric, densiometric, microstructural, and biomechanical properties of trabecular and cortical bone,^1–3^ contributing to fracture risk assessment.^4^^,^^5^ Furthermore, HR-pQCT data are used in the study of bone biology, disease progression, and treatment outcomes.^6–10^

The first-generation HR-pQCT scanner (XCTI) has an isotropic voxel size of 82 μm. At this resolution, XCTI image analysis relies on tissue density and histomorphometric model assumptions.^11^^,^^12^ The second-generation HR-pQCT scanner (XCTII) provides an improved voxel size of 61 μm. This higher resolution allows for direct measurement of all trabecular parameters using distance transform methods that do not rely on density and model assumptions.^13^ Higher resolution also provides improved visualization and quantification of fine features within both the trabecular and cortical compartments. However, the transition from first-generation to second-generation scanners poses challenges for longitudinal studies, multi-center trials, and the comparison of XCTII-derived data to historical XCTI-derived results, as these activities require ensuring compatibility and comparability of measurements across different scanners.

Cross-calibration is an established approach for determining relationships between measurements obtained from different devices, allowing for the estimation of measurements on newer devices using data collected from older or different devices.^14^^,^^15^ The objective of this study is to estimate second-generation HR-pQCT measurements from first-generation scanner data using cross-calibration. By establishing reliable cross-calibration equations, we aim to bridge the gap between different generations of HR-pQCT scanners and enable the utilization of the rich body of historical XCTI data in conjunction with the advanced capabilities of second-generation scanners. This approach holds promise for facilitating longitudinal studies, improving comparability across different research centers, and enhancing the clinical application of HR-pQCT in bone health assessment. In the following sections, we will describe the methodology employed for cross-calibration, present the validation results, and discuss the implications of our findings. We will perform cross-calibration using both the manufacturer’s standard XCTII Gaussian-based binarization approach, as well as the Laplace-Hamming (LH) segmentation approach we previously developed and validated for XCTII.^16^

## Materials and methods

### Study participants

Based on the sample size in our previously published study that highlighted the effect of LH segmentation approach on XCTII density and structural outcome metrics, 36 volunteers (16 women, 20 men; 56 ± 13 yr; age range 26–85; Table 1) were recruited to ensure sufficient eligible tibia and radius scans for cross-calibration.^17^ Women known to be pregnant or breast-feeding and men and women with metal implants at both scan sites (radius and tibia) were excluded. There were no other health- or bone-related inclusion or exclusion criteria for enrollment. The institutional review board at the University of California, San Francisco approved the study protocol, and all participants provided written informed consent prior to their involvement in the study.

**Table 1:** Participant characteristics categorized by Training and Test sets for bootstrapping.

**Characteristic**	**All participants (*n* = 36)**	**Training (*n* = 20)**	**Test (*n* = 16)**	***p*-value**
**Radius**				
**Age (yr)**	56 ± 13	55 ± 13	59 ± 13	.42
**Sex (*n*)**				
** Male**	20 (56%)	15 (58%)	5 (50%)	.68
** Female**	16 (44%)	11 (42%)	5 (50%)	
**Race (*n*)**				
** White**	26 (72%)	17	9	–
** Black/African American**	2 (6%)	2	0	
** Asian**	8 (22%)	7	1	
** Hispanic/Latino**	5 (14%)	4	1	
**Height (cm)**	171 ± 11	171 ± 11	171 ± 12	.99
**Weight (kg)**	76 ± 16	76 ± 15	77 ± 19	.91
**Body mass index (kg/m^2^)**	26 ± 4	26 ± 4	26 ± 6	.87
**Tibia**				
**Age (yr)**	56 ± 13	56 ± 14	56 ± 12	.93
**Sex (*n*)**				
** Male**	20 (56%)	10 (42%)	10 (83%)	.02
** Female**	16 (44%)	14 (58%)	2 (17%)	
**Race (*n*)**				
** White**	26 (72%)	17	9	–
** Black/African American**	2 (6%)	1	1	
** Asian**	8 (22%)	6	2	
** Hispanic/Latino**	5 (14%)	3	2	
**Height (cm)**	171 ± 11	170 ± 12	173 ± 9	.53
**Weight (kg)**	76 ± 16	72 ± 16	84 ± 14	.41
**Body mass index (kg/m^2^)**	26 ± 4	25 ± 4	28 ± 4	.39

Values are shown as mean ± SD or count (percentage). Unpaired student’s *t*-test and Pearson’s chi-squared test were performed between the Training and Test sets when applicable.

### Image acquisition

Volunteers were scanned on both first-generation (XCTI) and second-generation (XCTII) HR-pQCT scanners (Scanco Medical, Brüttisellen, Switzerland) using the manufacturer’s standard in vivo protocols. For XCTI, the scan settings were: source potential 60 kVp, tube current 900 mA, and isotropic 82-μm nominal resolution. Scans were acquired 9.5 and 22.5 mm proximal from the joint for distal radius and tibia, respectively, covering a length of 9.0 mm (110 slices). For XCTII, the scan settings were: source potential 68 kVp, tube current 1460 mA, and isotropic 61-μm nominal resolution. Scans were acquired 9 and 22 mm proximal from the joint for distal radius and tibia, respectively, covering a length of 10.2 mm (168 slices); compared with XCTI, XCTII captured ~0.5 mm more bone at either end of the scan volume. The non-dominant forearm and lower leg were scanned. In the case where a history of fracture or surgery was reported on the non-dominant side, the contralateral side was scanned. Prior to scanning and throughout the study, the scanners were routinely calibrated using scanner-specific quality control phantoms. All in vivo scans were visually inspected for motion artifacts using the manufacturer’s grading scheme to ensure adequate quality for all the images included for further analyses (grades 1–3).^18^

### Image analysis

XCTI images were analyzed using the manufacturer’s standard XCTI patient evaluation protocol, which involved semi-automated contouring of the periosteal and endosteal cortical boundaries, a LH filter and a fixed global threshold for binarization.^1^^,^^11^ The standard microstructural quantification scripts were applied, which use a combination of direct (eg, trabecular number) and indirect measurement techniques (eg, cortical thickness).

XCTII images were analyzed twice—first with the manufacturer’s standard Gaussian segmentation approach and second with the LH segmentation approach we adapted and validated in-house.^17^ In both XCTII analysis protocols, an auto-contouring process was first used to identify the periosteal and endosteal cortical boundaries; these auto-contours were checked and corrected manually if necessary. In the standard protocol (ie, the Gaussian segmentation approach), a Gaussian filter and fixed BMD thresholds were then used to extract the trabecular and cortical bone. In our developed LH segmentation approach, a LH filter followed by a fixed global threshold was used to extract the trabecular and cortical bone. In both analysis protocols, once the trabecular and cortical binary masks were created, direct measurement of all parameters was performed using distance transform methods.^13^ The LH approach affected only the microstructural parameters derived from the binary masks; volumetric density and geometry measurements remain identical for both the standard and LH analysis protocols.^17^ For both XCTI and XCTII, complete stacks were analyzed; no registration between XCTI and XCTII volumes was performed.

### Cross-calibration

To compare the agreement between the measurements obtained from the two scanners, a combination of cross-validation and bootstrapping approaches was utilized. For radius or tibia scans, the 36 data points were first randomly divided into two sets: one set with 12 data points and another with 24 data points. The 12-data points set was set aside for validation purposes (to determine the estimation error after cross-calibration), while the remaining 24 data points were used for cross-calibration (using linear regression analysis). During this step, 1000 sets of 12 data points were randomly selected (with replacement) for establishing cross-calibration equations, resulting in 1000 sets of slopes and intercepts for each output parameter (Table 1). The average values from these 1000 sets were recorded as the slope and intercept for that specific outcome measure.

Next for the validation step, the cross-calibration equations were applied to the first-generation output parameters to estimate the second-generation outputs, defined as XCTII^*^. To evaluate the accuracy of these estimates, the cross-calibration error was determined by comparing XCTII^*^ with the respective actual measured values on XCTII (ie, XCTII). This procedure was performed twice, once for the XCTII standard analysis approach and a second time for the XCTII LH approach.

### Statistical analysis

Participant demographics were compared between the training and test sets using unpaired student’s *t*-test and Pearson’s chi-squared test. Linear regression analysis was used to establish cross-calibration equations. Correlation strength was defined as strong (*R*^2^ > 0.9), moderate (0.7 < *R*^2^ < 0.9), and weak (*R*^2^ < 0.7). Bland–Altman plots were used to explore the differences between scanners. Statistical analyses were performed using JMP 16 software (SAS Institute Inc., Cary, NC, USA) with significance set at *p* <.05.

## Results

### Cross-calibration

Of the total 72 scans (36 radius, 36 tibia), 2 radius scans were excluded from analyses due to motion.

There were strong correlations between XCTI and XCTII density outcomes (Tt.BMD, Tb.BMD, Ct.BMD) at both radius and tibia (*R*^2^ > 0.88; Table 2). Bland–Altman plots showed good agreement for these outcomes between scanners at both sites although radius Ct.BMD was slightly underestimated by XCTII relative to XCTI (Figure S1; Note: the *R*^2^ values in Table 2 and the Supplementary Figures are different because Table 2 presents the average results from all 1000 bootstrapping iterations, while the Supplementary Figures present representative results from 1/1000 bootstrapping iterations).

**Table 2:** Regression analysis results for trabecular and cortical outcomes reported for both the standard and LH approach.

	**Standard**			LH		
	**Slope**	**Intercept**	***R*** ^**2**^	**Slope**	**Intercept**	***R*** ^**2**^
**Radius**						
** Tt.BMD**	1.03	−3.07	0.95	–	–	–
** Tb.Ar**	0.96	16.51	0.91	–	–	–
** Tb.BMD**	1.03	−14.20	0.99	–	–	–
** BV/TV**	1.77	−0.02	0.97	1.29	0.06	0.97
** Tb.N**	0.77	−0.11	0.76	0.74	0.18	0.63
** Tb.Th**	1.04	0.16	0.69	0.60	0.17	0.66
** Tb.Sp**	1.68	−0.06	0.88	1.18	0.04	0.79
** Tb.1/N.SD**	1.43	−0.01	0.92	1.10	0.01	0.95
** Ct.Ar**	0.95	5.50	0.97	–	–	–
** Ct.BMD**	1.16	−199.87	0.88	–	–	–
** Ct.Pm**	1.02	−1.71	0.96	–	–	–
** Ct.Po**	0.23	0.00	0.83	0.38	0.01	0.43
** Ct.Th**	0.99	0.15	0.92	–	–	–
** Ct.Po.Dm**	0.63	0.07	0.21	0.35	0.12	0.36
**Tibia**						
** Tt.BMD**	1.00	0.06	1.00	–	–	–
** Tb.Ar**	1.02	−4.24	0.99	–	–	–
** Tb.BMD**	1.04	−14.56	1.00	–	–	–
** BV/TV**	1.66	0.01	0.99	1.19	0.08	0.97
** Tb.N**	0.60	0.20	0.82	0.59	0.34	0.83
** Tb.Th**	1.16	0.16	0.31	0.61	0.17	0.48
** Tb.Sp**	1.28	0.14	0.79	1.11	0.12	0.83
** Tb.1/N.SD**	0.98	0.08	0.82	0.93	0.06	0.87
** Ct.Ar**	1.01	4.08	0.99	–	–	–
** Ct.BMD**	0.92	58.42	0.95	–	–	–
** Ct.Pm**	1.01	−0.77	1.00	–	–	–
** Ct.Po**	0.47	0.00	0.83	0.72	0.02	0.52
** Ct.Th**	1.12	0.06	0.99	–	–	–
** Ct.Po.Dm**	1.20	0.00	0.38	0.65	0.08	0.68

All values represent averages derived from the cross-calibration procedure. Abbreviation: LH, Laplace-Hamming.

Geometry outcomes Tb.Ar and Ct.Ar were strongly correlated between XCTI and XCTII at both the radius and tibia (*R*^2^ > 0.91; Table 2). Bland–Altman plots showed good agreement between scanners and proportional biases were small (Figure S2).

For trabecular microstructure outcomes, there were strong correlations between scanners for BV/TV at both the radius and tibia (*R*^2^ = 0.97 and 0.99, respectively; Table 2) and Tb.1/N.SD at radius (*R*^2^ = 0.92; Table 2). There were moderate correlations between scanners at both the radius and tibia for Tb.N (*R*^2^ = 0.76 and 0.82, respectively; Table 2) and Tb.Sp (*R*^2^ = 0.88 and 0.79, respectively; Table 2). Among all the outcomes, Tb.Th showed the weakest correlation between scanners at both the radius and tibia (*R*^2^ = 0.69 and 0.31, respectively; Table 2). Relative to XCTI, BV/TV, Tb.Th, Tb.Sp, and Tb.1/N.SD were overestimated by XCTII at both the radius and tibia, while Tb.N was underestimated by XCTII at both sites. Bland–Altman plots showed a strong proportional bias for BV/TV at both the radius and tibia, for Tb.Sp at the radius, and for Tb.N at the tibia (Figures S3 and S4). The analysis of XCTII data using our developed LH approach greatly reduced the observed proportionality bias (Figures S3 and S4).

For cortical microstructure outcomes, there was a strong correlation for Ct.Th at both radius and tibia between scanners (*R*^2^ > 0.92; Table 2). There was a moderate correlation for Ct.Po at both the radius and tibia between scanners (*R*^2^ = 0.83 at both sites; Table 2). Ct.Po.Dm showed the weakest correlations at both the radius and tibia (*R*^2^ = 0.21 and 0.38, respectively; Table 2). Relative to XCTI, Ct.Po was underestimated by XCTII at both the radius and tibia, while Ct.Po.Dm was overestimated by XCTII at both sites (Figures S5 and S6). There was a strong proportional bias in Ct.Po at both the radius and tibia, which was eliminated using our LH approach (Figures S5 and S6).

### Validation

**Figure 1:**
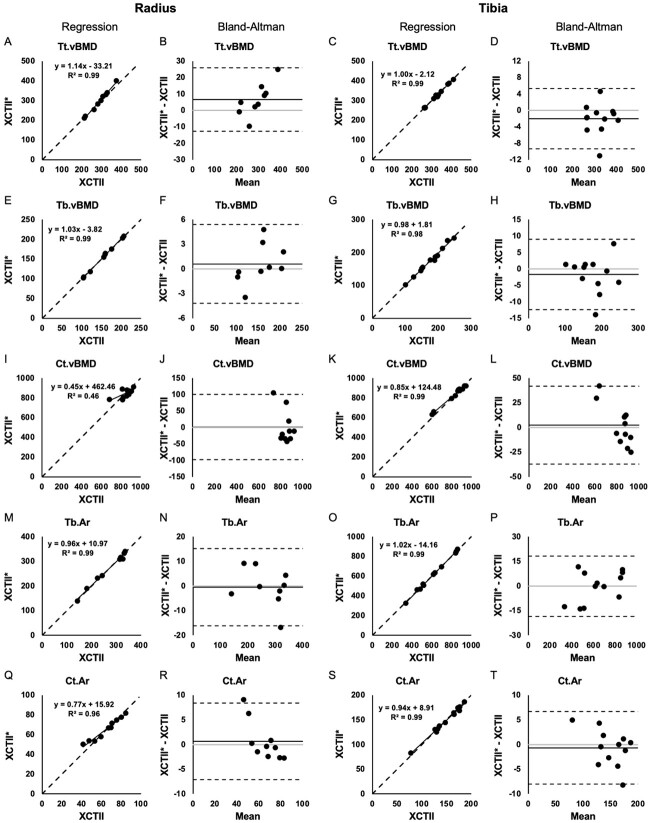
Regression and Bland–Altman plots for Tt.vBMD (A-D), Tb.vBMD (E-H), Ct.vBMD (I-L), Tb.Ar (M-P), and Ct.Ar (Q-T) assessed using measured XCTII values and the estimated XCTII^*^ values from XCTI at the radius (left) and tibia (right). On regression plots, the dashed line indicates the line of unity. On Bland–Altman plots, the solid black line indicates the mean difference, the dashed lines indicate the 95% limits of agreement, and the gray line indicates zero.

**Figure 2:**
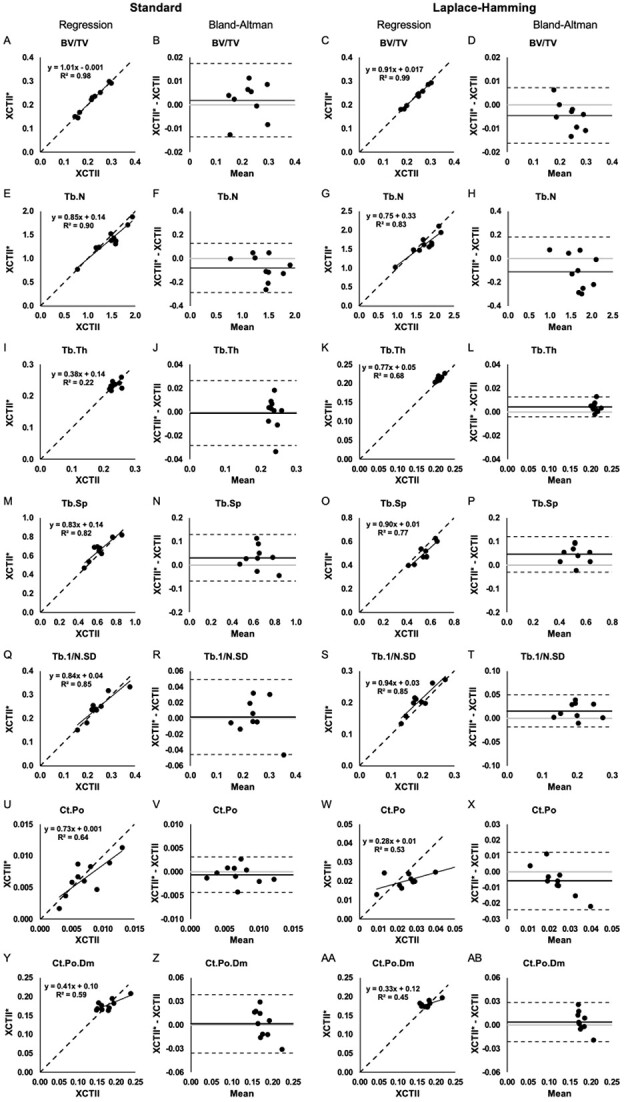
Regression and Bland–Altman plots for BV/TV (A-D), Tb.N (E-H), Tb.Th (I-L), Tb.Sp (M-P), Tb.1/N.SD (Q-T), Ct.Po (U-X), and Ct.Po.Dm (Y-AB) assessed using the measured XCTII values and the estimated XCTII^*^ values from XCTI at the radius using the standard (left) and LH (right) approach. On regression plots, the dashed line indicates the line of unity. On Bland–Altman plots, the solid black line indicates the mean difference, the dashed lines indicate the 95% limits of agreement, and the gray line indicates zero.

**Figure 3:**
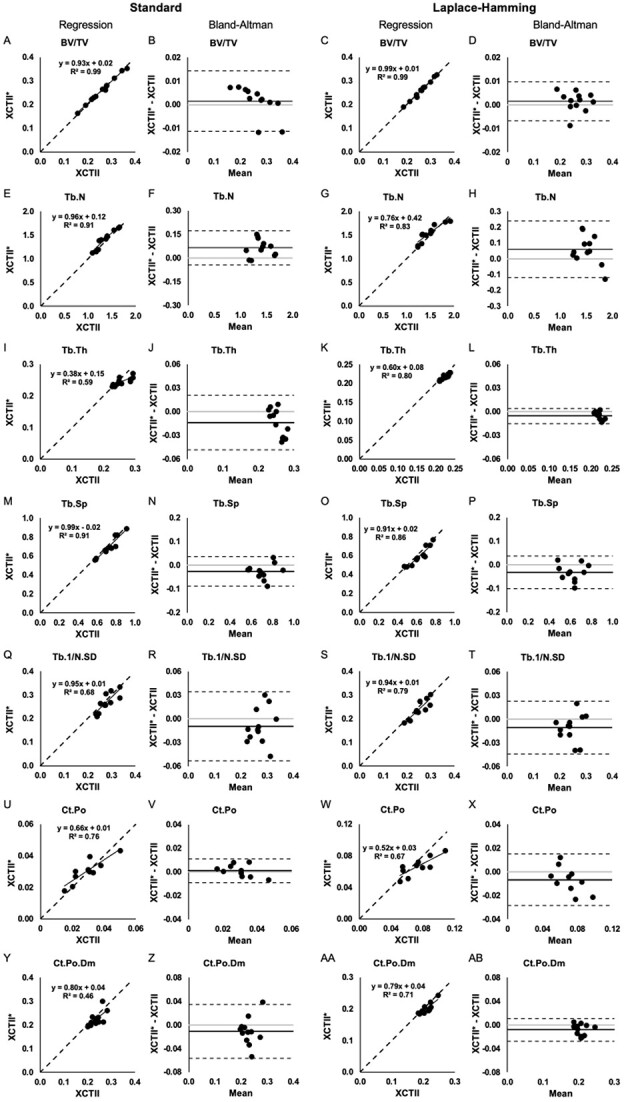
Regression and Bland–Altman plots for BV/TV (A-D), Tb.N (E-H), Tb.Th (I-L), Tb.Sp (M-P), Tb.1/N.SD (Q-T), Ct.Po (U-X), and Ct.Po.Dm (Y-AB) assessed using measured the XCTII values and the estimated XCTII^*^ values from XCTI at the tibia using the standard (left) and LH (right) approach. On regression plots, the dashed line indicates the line of unity. On Bland–Altman plots, the solid black line indicates the mean difference, the dashed lines indicate the 95% limits of agreement, and the gray line indicates zero.

Applying the established cross-calibration equations to our test set XCTI data, the estimated values for XCTII outcomes were calculated and compared against the measured values to compute percent error. Linear regression and Bland–Altman plots that compare measured XCTII vs. estimated XCTII^*^ outcomes using both standard and LH approaches are presented in Figures 1, 2, and 3.

For trabecular outcomes, the mean percent error was between 0.80% and 4.65% for density and geometry outcomes (Tt.BMD, Tb.BMD, Tb.Ar) and between 1.68% and 7.58% for microstructural outcomes (BV/TV, Tb.N, Tb.Th, Tb.Sp, Tb.1/N.SD). For cortical outcomes, the mean percent error was between 0.61% and 10.71% for density and geometry outcomes (Ct.BMD, Ct.Ar, Ct.Pm, Ct.Th) and between 6.81% and 21.98% for microstructural outcomes (Ct.Po, Ct.Po.Dm). Using the LH approach for cross-calibration resulted in smaller percent errors for BV/TV, Tb.Th, and Ct.Po.Dm, but larger percent errors for Tb.N, Tb.Sp, and Ct.Po (Table 3). More specifically, percent errors for Tb.Th from LH were significantly smaller at both the radius and tibia (*p* =.04 and <.001, respectively; Table 3); percent error for Ct.Po.Dm was significantly smaller at the radius (*p* =.006; Table 3).

**Table 3:** Mean absolute percent error between measured XCTII and estimated XCTII^*^ outcomes by cross-calibration for trabecular and cortical outcomes, reported for both the standard and developed LH approach.

	**Tt.BMD**	**Tb.Ar**	**Tb.BMD**	**BV/TV**	**Tb.N**	**Tb.Th**	**Tb.Sp**	**Tb.1/N.SD**
**Radius**								
** Standard**	3.85 (1.49, 6.22)	4.66 (−0.70, 10.01)	2.79 (−0.69, 6.27)	3.96 (1.23, 6.70)	6.13 (2.46, 9.81)	3.92 (1.12, 6.72)	7.36 (3.20, 11.52)	7.40 (3.70, 11.11)
** LH**	–	–	–	2.88 (0.45, 5.32)	8.26 (4.61, 11.90)	1.57 (0.64, 2.51)	9.58 (5.14, 14.03)	9.73 (4.14, 15.32)
*** p*-value**				.12	.07	.04	.09	.33
**Tibia**								
** Standard**	0.80 (0.24, 1.36)	1.43 (0.60, 2.25)	2.05 (0.76, 3.33)	1.68 (0.63, 2.73)	4.66 (2.32, 6.99)	5.71 (3.13, 8.29)	4.98 (3.06, 6.89)	7.58 (5.04, 10.11)
** LH**	–	–	–	1.23 (0.46, 2.01)	6.04 (3.06, 9.02)	1.81 (0.87, 2.75)	6.00 (3.52, 8.48)	6.47 (3.47, 9.47)
*** p*-value**				.46	.17	<.001	.27	.38
	**Ct.Ar**	**Ct.BMD**	**Ct.Pm**		**Ct.Po**	**Ct.Th**	**Ct.Po.Dm**	
**Radius**								
** Standard**	5.09 (0.18, 10.01)	4.85 (1.67, 8.02)	3.95 (0.48, 7.43)		21.97 (9.48, 34.47)	10.74 (-0.81, 22.29)	9.30 (5.42, 13.18)	
** LH**	–	–	–		32.31 (7.83, 56.79)	–	5.41 (2.10, 8.72)	
*** p*-value**					.32		.006	
**Tibia**								
** Standard**	2.11 (0.85, 3.37)	2.12 (0.78, 3.46)	0.61 (0.42, 0.81)		14.23 (9.95, 18.51)	2.55 (0.77, 4.33)	6.81 (2.94, 10.68)	
** LH**	–	–	–		18.50 (8.11, 28.89)	–	3.99 (1.94, 6.04)	
*** p*-value**					.35		.15	

The *p*-values are obtained from the paired student’s *t*-test performed between the percent errors from the standard and LH approach. Abbreviations: LH, Laplace-Hamming; XCTII, second-generation. Significance has been provided as p-values in the table.

## Discussion

The goal of this study was to explore the feasibility and accuracy of estimating second-generation HR-pQCT measurements from the first-generation scanner with cross-calibration using linear regression equations. For most density, geometry, and microstructural outcomes, cross-calibration accurately estimated and eliminated the differences between scanners. However, there were large estimation errors for some microstructural outcomes, notably cortical porosity.

As expected, and consistent with previous studies, cross-scanner correlations for density and geometry outcomes were stronger compared with those for microstructural outcomes.^14^^,^^15^ Density and geometry metrics are less sensitive to resolution and image artifacts (including motion artifacts) due to their quantification over larger scales, lending them greater stability across scanners. This is also in line with previous multi-center studies reporting a smaller variability in density and geometry measures across different XCTI scanners compared with microstructural measures.^19^^,^^20^ Conversion of density and geometry metrics from first-generation to second-generation scanners is therefore feasible.

We found large differences in the absolute values of some microstructural outcomes between the two scanners. Consistent with the findings of Agarwal et al. and Manske et al.,^14^^,^^15^ we found that XCTII overestimated BV/TV, Tb.Th, and Tb.Sp and underestimated Tb.N and Ct.Po relative to XCTI. This can be explained by the inherent differences in the acquisition and analysis approach of the two scanners, including differences in resolution, image analysis (filtering and thresholding), and quantitative analysis approach (XCTII direct vs. XCTI indirect). The standard XCTII analysis approach also obscures some fine features in both the trabecular and cortical compartments in the segmentation step and thickens larger trabeculae, therefore resulting in overestimated BV/TV, Tb.Th, and Tb.Sp and underestimated Tb.N and Ct.Po, as established in our previous publication.^17^ Despite these differences in absolute values, the correlations between the two scanners were strong for these parameters, explaining the relatively small percent error in estimated values. However, for some other microstructural outputs such as Tb.Th and Ct.Po.Dm, in addition to the large differences in absolute values, the correlations were weak between the two scanners, resulting in relatively larger percent errors in the estimated values. This could be due to the higher sensitivity of these outcomes to spatial resolution, suggesting that estimating XCTII data from XCTI data for such measures may not be recommended using XCTII standard analysis approach. Using our developed XCTII LH approach, which is more consistent with the analysis approach of XCTI, improved the correlations and, therefore, resulted in smaller estimate errors for these metrics. In addition, we found stronger cross-scanner correlations and better agreement between the measured and estimated values at the tibia compared with the radius due to fewer motion artifacts in the tibia scans compared with the radius scans.

Our study has a number of strengths. First, including both the standard and LH binarization approaches provides direct comparisons and insight into the effects of different segmentation approaches on the cross-calibration process and the consequent estimations. Next, combining cross-validation and bootstrapping takes advantage of the benefits of both two methods—bootstrapping provides estimates for the parameters, and cross-validation provides estimates of the test error.

Our study also has a number of important limitations. First, despite the proven improved accuracy and reproducibility of the LH binarization approach compared with the standard approach for XCTII for the cohort studied here, the effect of LH on HR-pQCT outcome metrics has not been examined for disease cohorts with pathology-specific bone microarchitecture (eg, great cortical bone loss with chronic kidney disease). Thus, the effect of LH should be evaluated on a cohort-by-cohort basis before any generalization can be made upon further validation. Additionally, although bootstrapping was deployed to minimize the cross-calibration bias, sex was not matched between the tibia training and test sets, potentially contributing to the proportional biases and limiting the robustness of the cross-calibration results when extrapolated to larger and more diverse cohorts. However, this bootstrapping approach can be readily adapted to produce additional cross-calibration equations in diverse cohorts, which can potentially be integrated with our cross-calibration equations with improved generalizability. Last, it is worth mentioning that since we chose to perform our analysis on full scan regions for both XCTI and XCTII, and reference line placement inconsistency may be a potential contributor to our reported error values. However, all our scans were acquired by one skilled operator to minimize such errors.

In summary, we found good agreement between density and geometry outcomes measured by XCTI and XCTII; there were large differences in the absolute values for some of the microstructural outcomes, suggesting that they should be directly compared between the two scanners. However, we found moderate to strong correlations for these microstructural outcomes between scanners despite the large differences in absolute values, showing that XCTII outcomes can be estimated from XCTI measurements using the established cross-calibration equations. For microstructural outputs such as Tb.Th and Ct.Po.Dm that have a higher sensitivity to spatial resolution, the standard segmentation approach on XCTII resulted in weak correlations between the scanners, while the LH approach we developed and validated in-house resulted in stronger correlations between the two scanners for these outcomes. These stronger correlations were accompanied by reduced percent errors in these outcomes, as well decreased differences in absolute values and the proportional bias in Bland–Altman plots for other measurements. For these reasons, and considering the improved accuracy of our LH approach compared with the standard approach established in our previous publication,^17^ we propose that investigators should use the LH approach for analyzing scans on XCTII, particularly when comparing with XCTI data.

## Supplementary Material

XCalib_revision_main_manuscript_FINAL_GJK_clean_081224_SUP

## Data Availability

The data that support the findings of this study are available on request from the corresponding author. The data are not publicly available due to privacy or ethical restrictions.
