# Supplementary material for: HR-pQCT cross-calibration using standard vs. Laplace-Hamming binarization approach
Source: JBMR Plus. 2024 Aug 27;8(10):ziae116. doi: 10.1093/jbmrpl/ziae116 (PMC11417609; doi:10.1093/jbmrpl/ziae116)
Supplement: XCalib_revision_main_manuscript_FINAL_GJK_clean_081224_SUP [file xcalib_revision_main_manuscript_final_gjk_clean_081224_sup.docx]

# **Supplemental Materials**


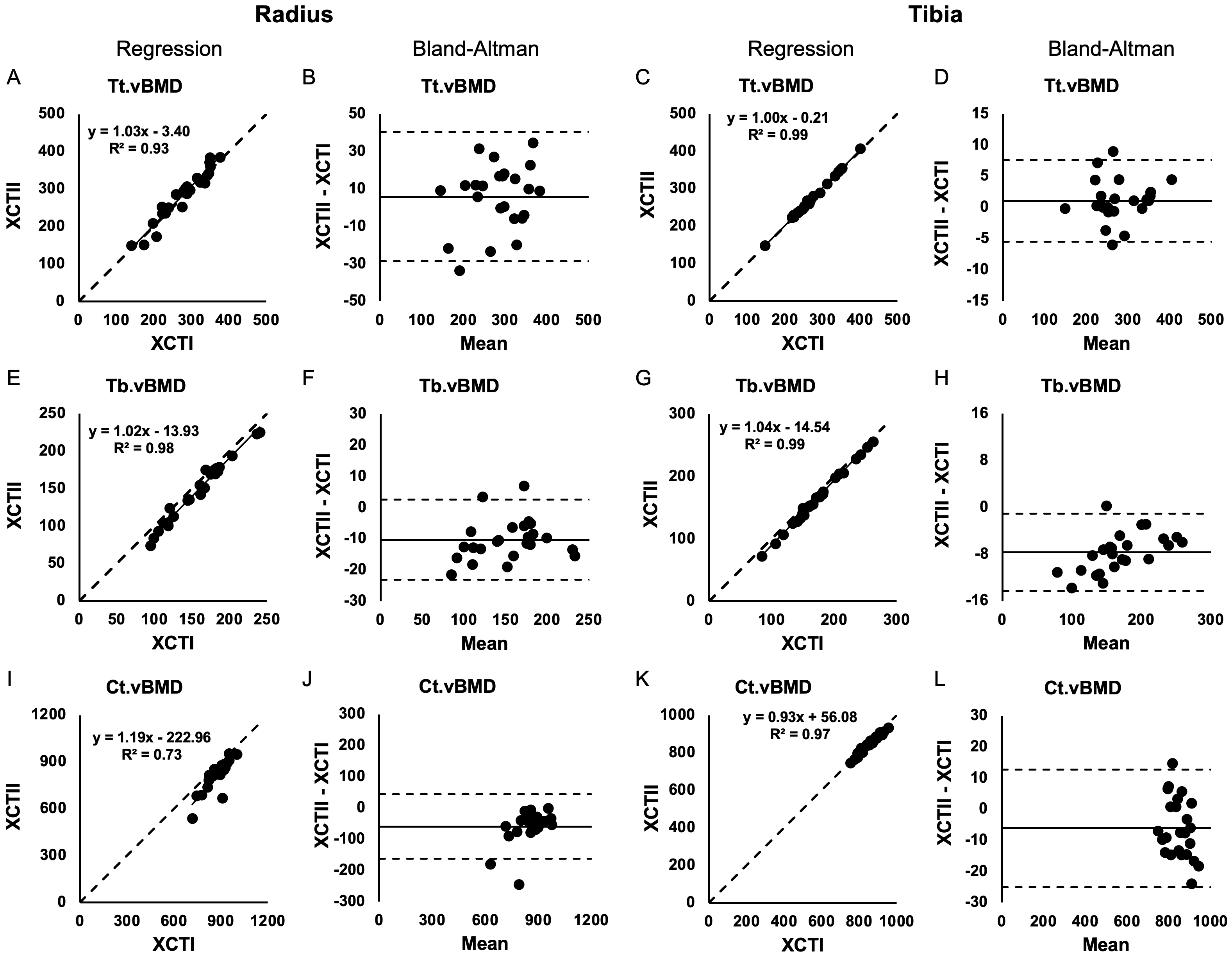


**Figure S1.** Regression and Bland-Altman plots for Tt.BMD **(A-D)**, Tb.BMD **(E-H)**, and Ct.BMD **(I-L)** assessed using the standard XCTII approach for cross-calibration at the radius (left) and tibia (right). On regression plots, the dashed line indicates the line of unity. On Bland-Altman plots, the solid line indicates the mean difference, and the dashed lines indicate the 95% limits of agreement. All subplots are representative results from one bootstrapping iteration.
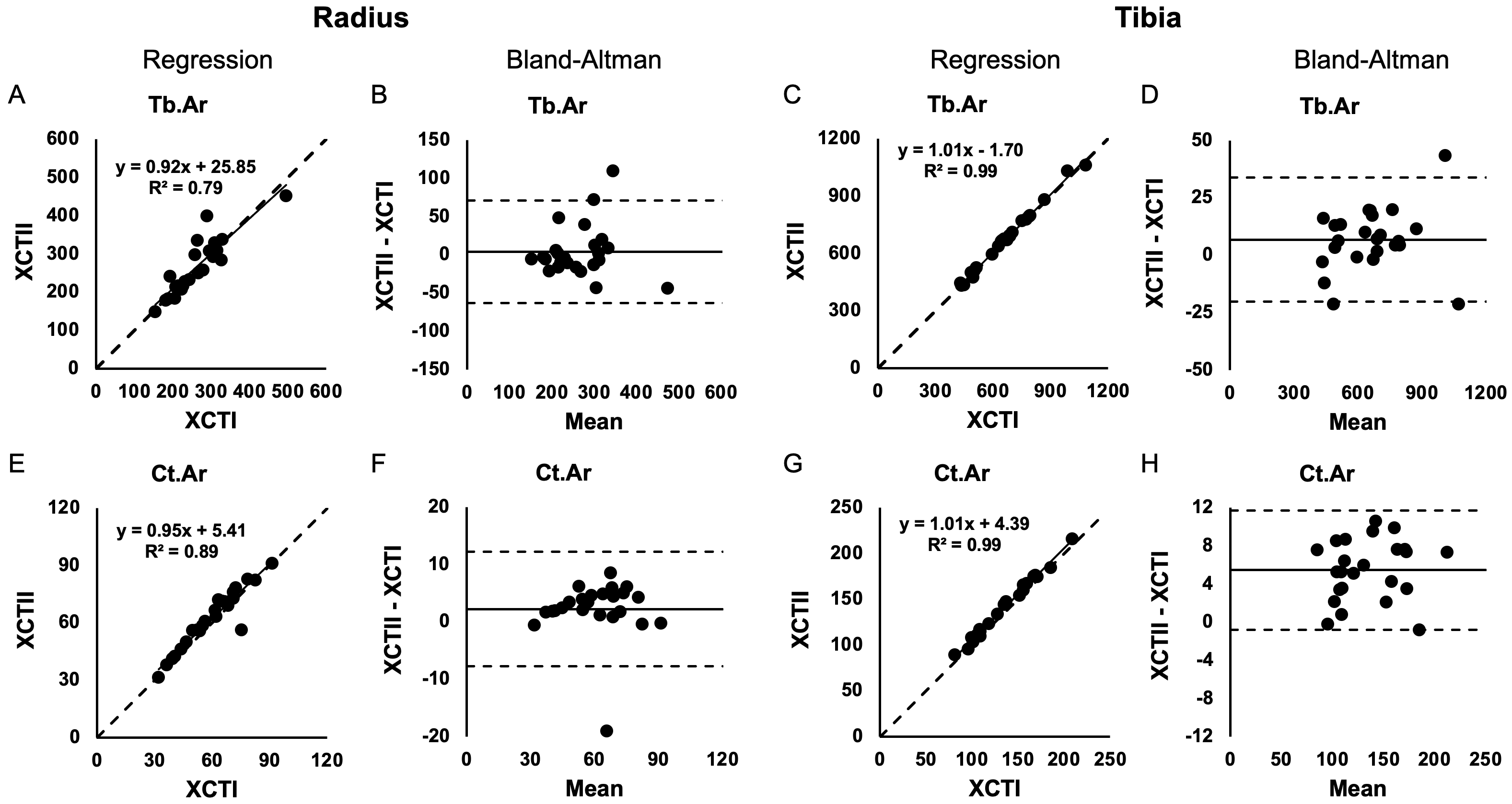


**Figure S2.** Regression and Bland-Altman plots for Tb.Ar **(A-D)**, and Ct.Ar **(E-H)** assessed using the standard XCTII approach for cross-calibration at the radius (left) and tibia (right). On regression plots, the dashed line indicates the line of unity. On Bland-Altman plots, the solid line indicates the mean difference, and the dashed lines indicate the 95% limits of agreement. All subplots are representative results from one bootstrapping iteration.


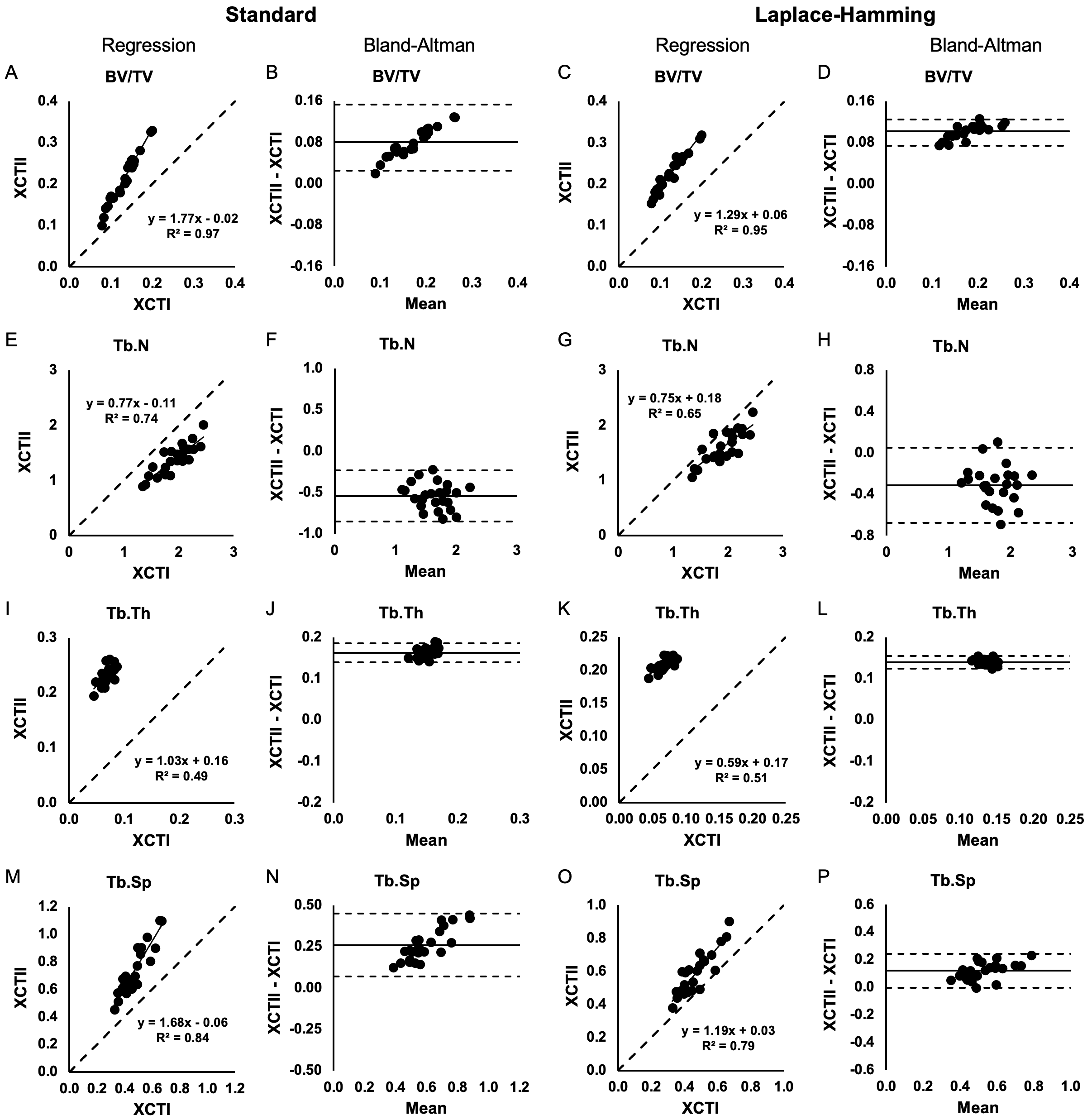


**Figure S3.** Regression and Bland-Altman plots for BV/TV **(A-D)**, Tb.N **(E-H)**, Tb.Th **(I-L)**, and Tb.Sp **(M-P)** assessed using the standard XCTII and LH approach for cross-calibration at the radius. On regression plots, the dashed line indicates the line of unity. On Bland-Altman plots, the solid line indicates the mean difference, and the dashed lines indicate the 95% limits of agreement. All subplots are representative results from one bootstrapping iteration.


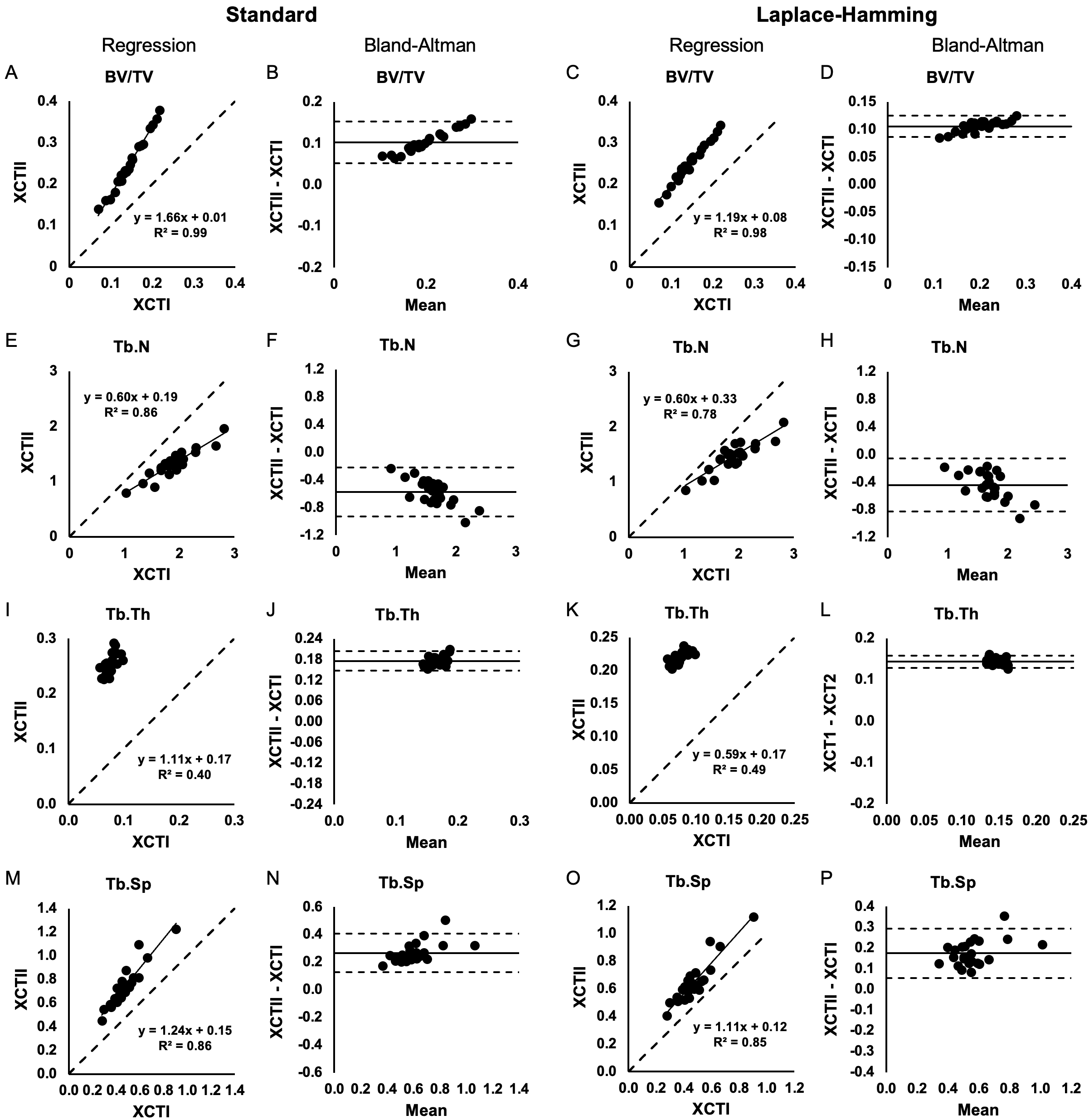


**Figure S4.** Regression and Bland-Altman plots for BV/TV **(A-D)**, Tb.N **(E-H)**, Tb.Th **(I-L)**, and Tb.Sp **(M-P)** assessed using the standard XCTII and LH approach for cross-calibration at the tibia. On regression plots, the dashed line indicates the line of unity. On Bland-Altman plots, the solid line indicates the mean difference, and the dashed lines indicate the 95% limits of agreement. All subplots are representative results from one bootstrapping iteration.


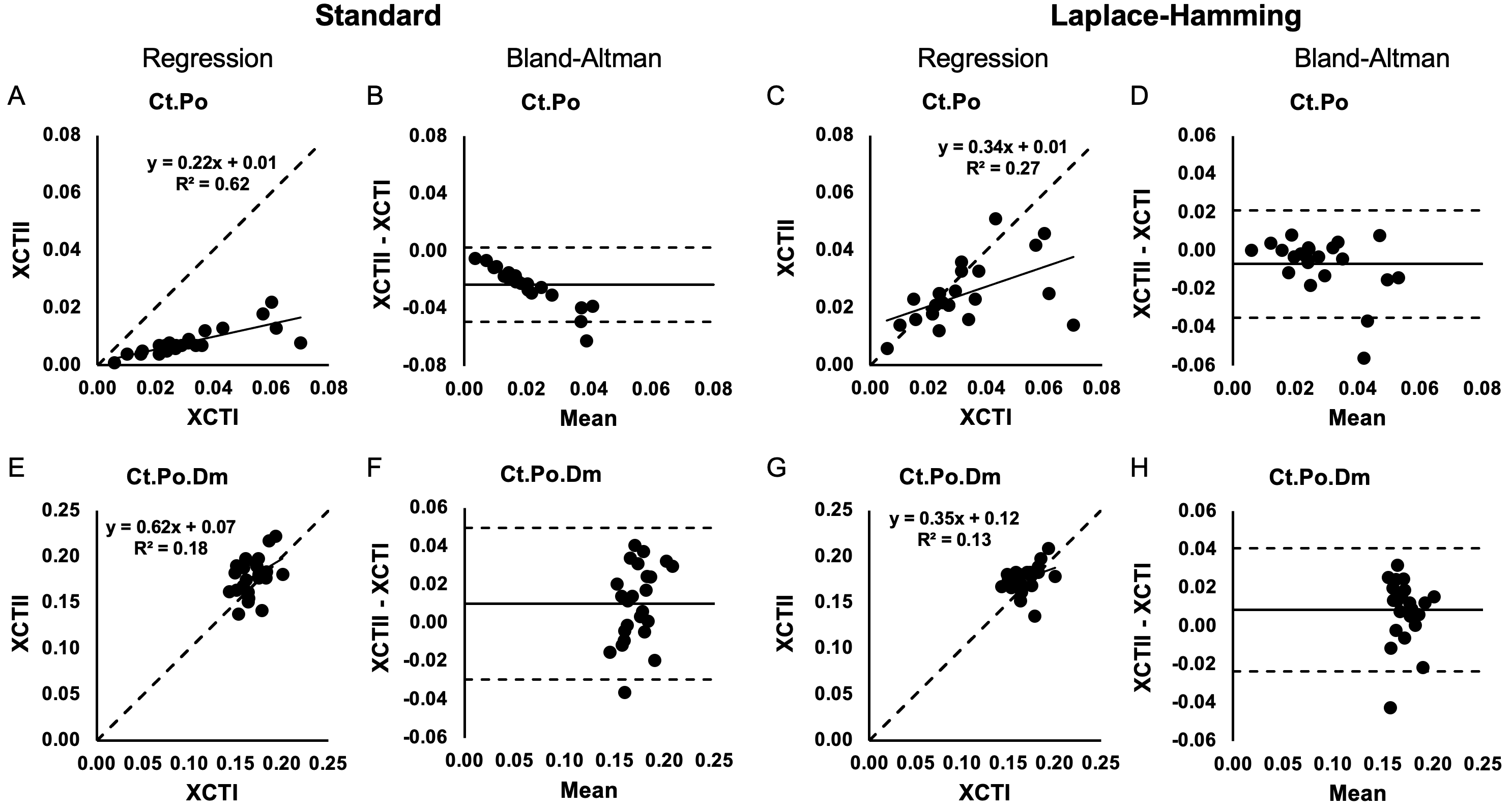


**Figure S5.** Regression and Bland-Altman plots for Ct.Po **(A-D)**, and Ct.Po.Dm **(E-H)** assessed using the standard XCTII and LH approach for cross-calibration at the radius. On regression plots, the dashed line indicates the line of unity. On Bland-Altman plots, the solid line indicates the mean difference, and the dashed lines indicate the 95% limits of agreement. All subplots are representative results from one bootstrapping iteration.


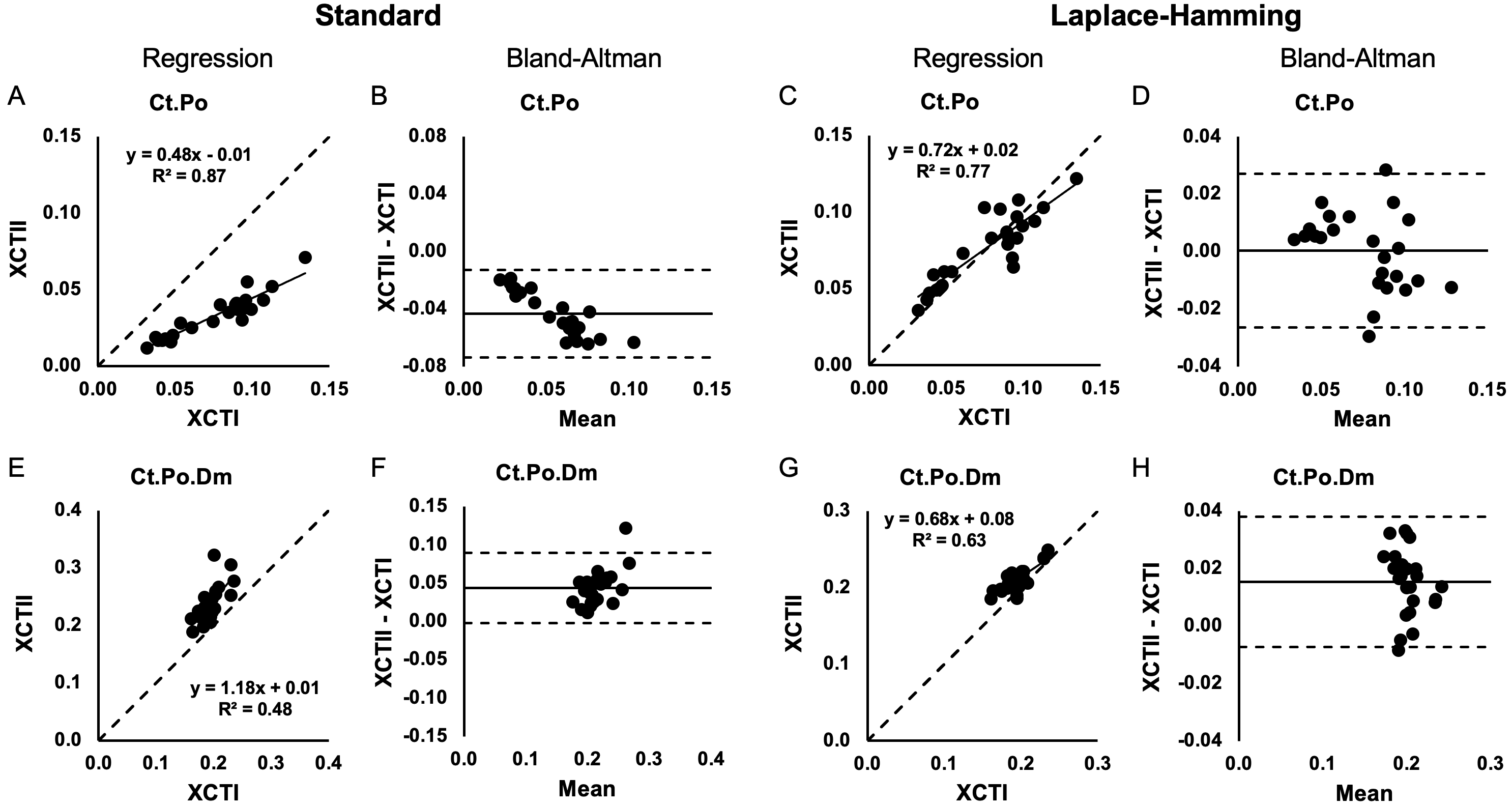


**Figure S6.** Regression and Bland-Altman plots for Ct.Po **(A-D)**, and Ct.Po.Dm **(E-H)** assessed using the standard XCTII and LH approach for cross-calibration at the tibia. On regression plots, the dashed line indicates the line of unity. On Bland-Altman plots, the solid line indicates the mean difference, and the dashed lines indicate the 95% limits of agreement. All subplots are representative results from one bootstrapping iteration.
